# Supplementary material for: A Novel Fluorescent Sensor Based on Aptamer and qPCR for Determination of Glyphosate in Tap Water
Source: Sensors (Basel). 2023 Jan 6;23(2):649. doi: 10.3390/s23020649 (PMC9863111; doi:10.3390/s23020649)
Supplement: Supplementary file 1 [file sensors-23-00649-s001.zip › sensors-2099815-supplementary.pdf]

Supporting Information

A Novel Fluorescent Sensor Based on Aptamer and qPCR for Determination of Glyphosate in Tap Water

Yong Shao [1,2,†], Run Tian [1,2,†], Jiaqi Duan [3], Miao Wang [1,2,*], Jing Cao [1,2], Zhen Cao [1,2], Guangyue Li [3], Fen Jin [1,2], A. M. Abd El-Aty [4,5,6] and Yongxin She [1,2,*]

1 Institute of Quality Standardization & Testing Technology for Agro-Products, Chinese Academy of Agricultural Sciences, Beijing 100193, China
2 Key Laboratory of Agrofood Safety and Quality (Beijing), Ministry of Agriculture and Rural Areas, Beijing 100081, China
3 State Key Laboratory for Biology of Plant Diseases and Insect Pests, Institute of Plant Protection, Chinese Academy of Agricultural Sciences, Beijing 100193, China
4 State Key Laboratory of Biobased Material and Green Papermaking, Shandong Academy of Sciences, Qilu University of Technology, Jinan 250353, China
5 Department of Pharmacology, Faculty of Veterinary Medicine, Cairo University, Giza 12211, Egypt
6 Department of Medical Pharmacology, Medical Faculty, Ataturk University, 25240 Erzurum, Turkey
* Correspondence: wm0510@126.com (M.W.); 0891syx@163.com (Y.S.)
† These authors contributed equally to this work.

Table S1 Sequences of Oligonucleotides Used in This Work

Oligo	Sequence(5′-3′)
polyA	AAAAAAA-NH_2
polyT-Aptamer[1]	TTTTTTTGCTAGACGATATTCGTCCATCCGAGCCCGTGGCGGGCTTTAGGACTCTGCGGGCTTCGCGGCGCTGTCAGACTGAATATGTC
complementary DNA	GACATATTCAGTCTGACAGCGCCGCGAAGCCCGCAGAGTACTAAAGCCCGCCACGGGCTCGGATGGACGAATATCGTCTAGC
upstream primer	GCTCGGATGGACGAATATCGTCTAG
downstream primer	TATTCGTCCATCCGAGCCCGTGGCG

Table S2 qPCR conditions

95℃ 30 S

95℃ 5 S

60℃ 34 S — 40 circles

95℃ 15 S

60℃ 1 min

Table S3 Estimation of glyphosate recovery based on this method

Sample	Standard Addition Amount (ppm)	Recovery Rate(%)			RSD(%)
Tap water	1.4	105.4	96.1	113.1	0.58
	0.7	109	96.9	104.3	0.40
	0.35	104.9	91.5	114.4	0.73

References

1. Chen, F.; Li, G.; Liu, H.; Leung, C.-H.; Ma, D.-L., G-quadruplex-based detection of glyphosate in complex biological systems by a time-resolved luminescent assay. *Sensors and Actuators B: Chemical* **2020,** *320*.
